# Clinicopathological Significance and Potential Drug Target of CDKN2A/p16 in Endometrial Carcinoma

**DOI:** 10.1038/srep13238

**Published:** 2015-08-18

**Authors:** Li Su, Hanwei Wang, Jingwei Miao, Ying Liang

**Affiliations:** 1 Department of Gynecologic Oncology, Beijing Obstetrics and Gynecology Hospital, Capital Medical University, Beijing 100006, China; 2 Nanjing Normal University, Nanjing 210097; 3 Nanjing University, Nanjing 210023.

## Abstract

Previous studies demonstrated that the loss of function of the *CDKN2A*/*p16/INK4A* gene is mainly caused by the hypermethylation of *CDKN2A*, however, whether or not it is associated with the incidence and clinicopathological characteristics of endometrial carcinoma (EC) remains unclear. In this study, we conducted a meta-analysis aiming to comprehensively assess the role of *CDKN2A* hypermethylation in the pathogenesis of EC. A detailed literature search was made to identify the related research publications. Analysis of pooled data was performed. Odds ratio (OR) was calculated and summarized. Final analysis of 638 EC patients from 12 eligible studies was performed. The results showed that *CDKN2A* hypermethylation was significantly higher in EC than in normal control tissue, the pooled OR from 8 studies including 400 EC patients and 131 controls, OR = 8.39 with 95% CI 4.03–17.45, test for overall effect, Z = 5.69, P < 0.00001. Further analysis showed that *CDKN2A* hypermethylation was not significantly associated with tumor differentiation and clinical stage status in EC patients. The results of this meta-analysis suggest that *CDKN2A* hypermethylation may be implicated in the pathogenesis of EC. *CDKN2A* hypermethylation was not significantly associated with tumor differentiation and clinical stage status in EC patients, indicating that *CDKN2A* hypermethylation might be early event of EC.

Endometrial carcinoma (EC) (also referred to as corpus uterine cancer or corpus cancer) is the most frequently occurring female genital cancer in the Western world, with 52,630 new cases and 8590 deaths expected in the United States alone in 2014^1^. To date, around 30% of EC patients are still diagnosed at later stages, most clinical trials of chemotherapeutics for advanced and recurrent EC have shown limited benefits and the incidence and mortality rate of EC have dramatically increased in the past few years^2^,^3^,^4^. Therefore, investigation of the mechanism of initiation, progression, prognostic markers and therapeutic targets is still needed for the selection of patients with EC and to provide better individualized treatment. Epigenetic modification of gene expression plays critical roles in carcinogenesis. Aberrant methylation of CpG dinucleotides is one of the commonly observed epigenetic modifications in human cancer^5^,^6^,^7^. Thus, the analysis of specific gene methylation as a tool for diagnosis of tumors or its use as prognostic marker has been widely used for many different cancers including EC^8^,^9^,^10^.

Tumor suppressor *CDKN2A*/*p16/INK4A* gene is located on chromosome 9p21, a cell cycle-related gene, belongs to the cdkn2 cyclin-dependent kinase inhibitor family, is one of the crucial defenses against cancer development in number of human cancers^11^,^12^. A large body of evidence suggests that *CDKN2A* is a target of inactivation in EC. In addition to mutation and homozygous deletions, frequent 5′-CpG island methylation of *CDKN2A* gene resulting in transcriptional silencing of this gene is noted as an important event in the development of EC. Previous studies have demonstrated that the inactivation of *CDKN2A* gene is mainly caused by its promoter and/or exon 1 hypermethylation in EC, however, the association of clinicopathological significance between *CDKN2A* hypermethylation and EC remains under investigation^13^. In addition, the reported methylation rates of *CDKN2A* in EC are remarkably diverse. Therefore, we performed this meta-analysis to investigate the effects of *CDKN2A* hypermethylation on the incidence and major clinicopathological features of EC.

## Methods

### Data source and search

We searched electronic databases including PubMed (1966 to December 2014), Web of Science (1945 to December 2014), EMBASE (1980 to December 2014). The keywords used were “CDKN2A”, “p16”, “p16^INK4a^”, “methylation”, “endometrial cancer”, “endometrial carcinoma” and “clinical studies”. Articles identified through the above search approach were screened by titles first, then by abstracts of the publications. All clinical studies except case reports were chosen, for instance, randomized controlled trials (RCTs), cohort studies, case-controls studies and case series. The language of publication was restricted to English and Chinese. All searched data were retrieved. Authors’ bibliographies and references of selected studies were also searched for other relevant studies. The most complete study was chosen to avoid duplication if same patient populations were reported in several publications. Studies meeting the following inclusion criteria were included: (1) *CDKN2A* methylation and/or expression which were evaluated in endometrial tissues, (2) researches which revealed the relationship between *CDKN2A* methylation and/or expression and endometrial cancer clinicopathological parameters and prognosis, (3) *CDKN2A* methylation and/or expression which were examined by methylation specific polymerase chain reaction (MSP), (4) articles which were published as a full papers in English or Chinese, (5) articles which provided sufficient information to estimate hazard ratio (HR) about overall survival and 95% confidence interval (CI) and probabilities for overall survival where applicable. The exclusion criteria included the following: (1) studies without control tissues including normal endometrium or non-tumor tissues; (2) letters, reviews, case reports, conference abstracts, editorials, expert opinion; (3) articles having no information on overall survival or those that could not calculate the HR about overall survival from the given information; and (4) all publications regarding *in vitro*/*ex vivo* studies, cell lines and human xenografts were also excluded.

### Data extraction

Two investigators independently extracted data from eligible studies. Disagreements were resolved by discussion till consensus achieved. Two investigators reviewed all of the articles that fit inclusion and exclusion criteria. The following information was recorded for each study: the name of first author, year of publication, sample source, number of cases, clinicopathological parameters, stage, *CDKN2A* methylation and/or expression, and patient survival. Data for study characteristics and clinical response were summarized and the data were turned into table format. Heterogeneity of investigation was evaluated to determine whether the data of the various studies could be analyzed in a meta-analysis.

### Statistical analysis

Analysis was conducted using the Stata 12.0 (Stata Corporation, TX, USA) and Review Manager 5.2 (Cochrane Collaboration, Oxford, UK). Comparisons of dichotomous measures were done by pooled estimates of odds ratios (ORs) as well as their 95% CIs. P value of <0.05 was considered to be statistically significant. Heterogeneity was examined by a chi-square test with significance being set at P < 0.10; the total variation among studies was estimated by I square. We used I square statistic to assess heterogeneity. The I square value is an estimate of variance due to between-study heterogeneity rather than chance (the Cochran Q statistics). Substantial heterogeneity exists when I square exceeding 50%. If there was heterogeneity among studies, we used a random effect model to pool the ORs; otherwise, a fixed effect model was selected.

Publication bias was assessed using an approach reported by Egger *et al.*^14^. In addition, we also checked reasons for statistical heterogeneity using meta-regression and sensitivity analysis.

## Results

**Figure 1:**
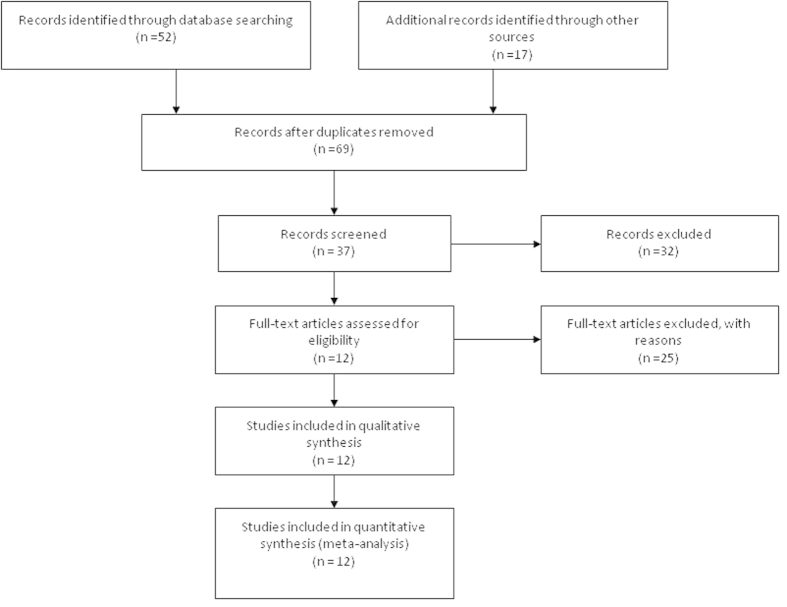
Flow chart of the study selection.

The meta-analysis was included 12 studies and a total of 638 patients after screening 69 articles by reviewers (Fig. 1). The following items were collected from each study: first author, published year, geographical location, pathological status, clinical stage status and *CDKN2A* methylation status as well as detective methods of *CDKN2A* methylation (Table 1). Twelve selected articles were checked and evaluated. High levels of methodological quality (>6 stars) were observed according to Newcastle-Ottawa quality assessment scale^15^.

**Figure 2:**
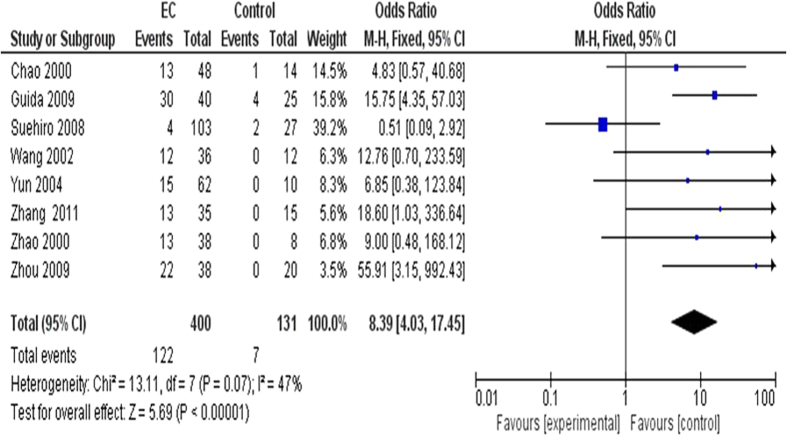
The pooled OR from 8 studies, including 400 EC patients and 131 controls. The pooled OR was 8.39 with 95% CI 4.03–17.45, test for overall effect, Z = 5.69, P < 0.00001.

Eight studies with a total of 400 EC patients and 131 controls with *CDKN2A* gene hypermethylation status were analyzed. Since the studies showed no significant heterogeneity (*I*^2^ = 47%), a fixed effect model was selected and used in current analysis. The pooled OR was 8.39 with 95% CI 4.03–17.45, test for overall effect, Z = 5.69, P < 0.00001, indicating that *CDKN2A* gene hypermethylation was significantly correlated with the EC patients (Fig. 2).

**Figure 3:**
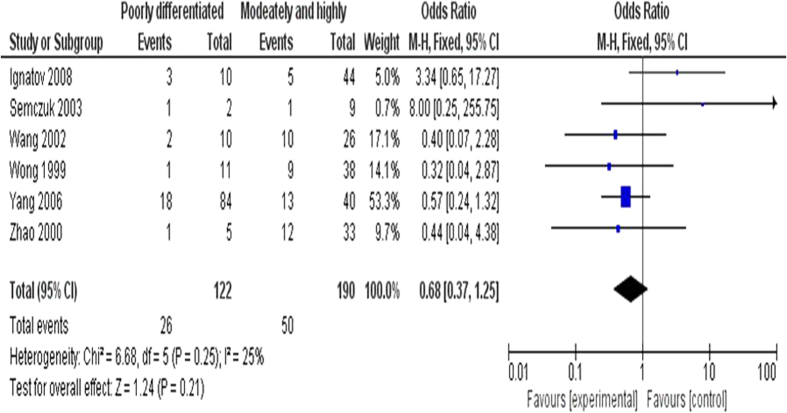
Aberrant *CDKN2A* hypermethylation was not significantly higher in poorly differentiated EC than that in moderately or highly differentiated EC, OR = 0.68, 95% CI = 0.37–1.25, *p* = 0.21.

**Figure 4:**
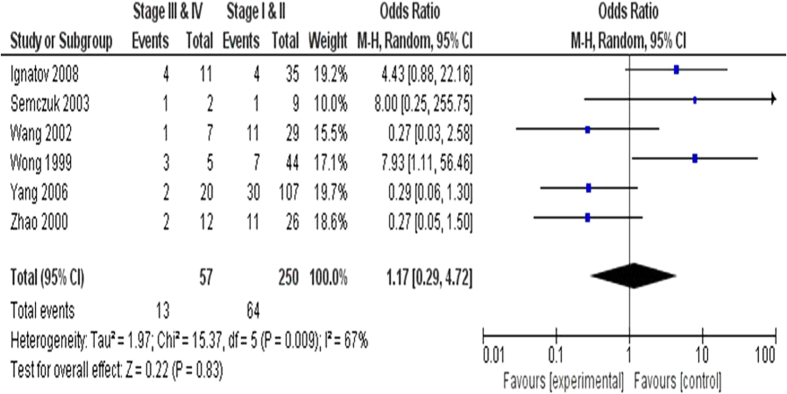
Aberrant *CDKN2A* hypermethylation was also not significantly higher in advanced EC (III & IV) than that in early staged EC (I & II), OR = 1.17, 95% CI = 0.29–4.72, *p* = 0.83.

We further analyzed 312 EC patients pooled from 6 studies to assess whether or not the aberrant *CDKN2A* hypermethylation in EC was associated with the differentiated status. As shown in Fig. 3, aberrant *CDKN2A* hypermethylation was not significantly higher in poorly differentiated EC than that in moderately and highly differentiated EC, OR = 0.68, 95% CI = 0.37–1.25, *p* = 0.21. In addition, we analyzed 307 EC patients pooled from 6 studies to assess whether or not the aberrant *CDKN2A* hypermethylation in EC was associated with the clinical stage status. Aberrant *CDKN2A* hypermethylation was observed to be not significantly higher in advanced EC (III & IV) than that in early staged EC (I & II), OR = 1.17, 95% CI = 0.29–4.72, *p* = 0.83, Fig. 4. These results suggest that *CDKN2A* hypermethylation may not play an important role in EC progression and different stages.

**Figure 5:**
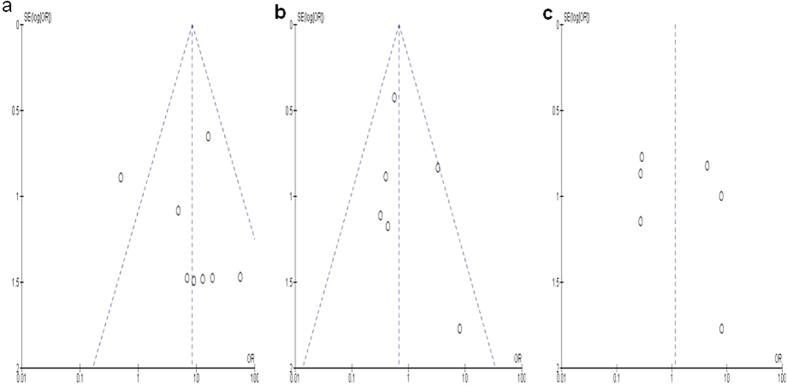
The funnel plots were largely symmetric, suggesting that there were no publication biases in the meta-analysis of *CDKN2A* hypermethylation in the EC patients. The funnel plot from 8 studies comparing EC and control tissues (**a**) comparing poorly differentiated EC and moderately or highly differentiated EC (**b**) comparing advanced EC (III & IV) and early staged EC (I & II) (**c**). X axis: value of Odds ratio (OR); Y axis: Standard errors (SE) multiply log scale of OR.

A sensitivity analysis, in which one study was removed at a time, was conducted to assess the result stability. The pooled OR was not significantly changed, indicating the stability of our analyses. The funnel plots were largely symmetric (Fig. 5) suggesting that there were no publication biases in the meta-analysis.

## Discussion

*CDKN2A* gene hypermethylation are common epigenetic aberrations in several type of cancer, including EC. In fact, *CDKN2A* gene mutations are rarely examined in patients of EC^16^,^17^. For example, one study determined only one point mutation in 36 cases of EC patients^18^. *CDKN2A* homozygous deletions were also rarely reported in EC patients. *CDKN2A* homozygous deletions were determined in only 1 of out 38 EC patients (3%)^16^ and no *CDKN2A* deletions in all of 36 EC patients in another study^18^. Since the reported methylation rates of *CDKN2A* in EC are remarkably diverse, the role of *CDKN2A* gene hypermethylation specific to the EC etiology remains elusive.

We conducted meta-analysis to determine the correlation between *CDKN2A* hypermethylation and clinicopathological characteristics in EC. Analysis of the pooled data showed that 1) EC had a higher hypermethylation than normal endometrial tissue; 2) *CDKN2A* hypermethylation was not significantly higher in poorly differentiated EC than that in moderately or highly differentiated EC; 3) In addition, *CDKN2A* hypermethylation was also not significantly higher in advanced EC (III & IV) than that in early staged EC (I & II). The results from the current study demonstrated that the hypermethylation rate of *CDKN2A* in EC was significantly higher than that in the normal endometrial tissues, indicating that *CDKN2A* hypermethylation was common in EC and *CDKN2A* gene hypermethylation could play an important role in endometrial carcinogenesis. The results from the current study indicate that the hypermethylation frequency of *CDKN2A* in EC is strongly associated with EC incidence, however, *CDKN2A* hypermethylation may be an early event in carcinogenesis of EC. Thus, detection of *CDKN2A* hypermethylation in EC may provide a valuable maker for prediction of early endometrial carcinogenesis. Since changes in *CDKN2A* hypermethylation are reversible, drug treatment through demethylation may be useful to delay carcinogenesis and progression. In fact, the effects of 5-aza-2-deoxycytidine (AZA) on tumor growth inhibition were reported on human EC xenografted in nude mice and the inhibition rates of the tumor were even reached to 79.10% in AZA treatment (P < 0.01)^19^. These preclinical studies strongly suggest the therapeutic potential of restoration of tumor suppressor expression through epigenetic modulation. This approach may bring new direction and hope for cancer treatment through gene-targeted therapy in future study.

Consistent results were obtained by sensitivity analyses, and no evidence of publication bias was found. However, this study has several limitations. There are many factors could affect methlation status and did not considered in our meta-analysis due to limited information. For example, the age of patients is one of the factors. Age-related changes in a biochemical process, such as DNA methylation could be responsible for the increased risk of cancer in older men and women^20^, unfortunately, the studies did not provide enough age-related information for our further analysis. In the comparison of tumor to control the study from Japan by Suehiro^21^ is a clear outlier from all other studies. The heterogeneity comes from this study, since *I*^*2*^ will get 0 and the pooled OR will be 12.07 with 95% CI 5.12–28.45, if without this study for meta-analysis of other 7 studies. One of possible reasons is that they failed to pick up correct primers for detection of *CDKN2A* hypermethylation status. In the comparison of *CDKN2A* hypermethylation with the differentiated status of EC, we determined the pooled OR from the two european studies and the four east studies, respectively. The pooled OR will be 3.92 with 95% CI 0.9–17.01 from the two european studies^22^,^23^, while the pooled OR will be 0.49 with 95% CI 0.25–0.96 from the four east studies^24^,^25^,^26^,^27^. These results indicate that *CDKN2A*hypermethylation may have different effect in moderately and highly differentiated EC from the european and far east countries. The search strategy in this meta-analysis was restricted to publications in English and Chinese. Articles published in other languages were not selected for meta-analysis, because of the difficulties in obtaining accurate medical translation. The possibility of selection biases could not be completely excluded, since all of the included studies were observational. Therefore, cautions should be taken when our findings are interpreted among the general populations.

In summary, in the present study, we report that *CDKN2A* hypermethylation was correlated with an increased risk of EC. *CDKN2A* hypermethylation was not significantly associated with tumor differentiation and clinical stage status in EC patients, indicating that *CDKN2A* hypermethylation might be early event of EC carcinogenesis.

## Additional Information

**How to cite this article**: Su, L. *et al.* Clinicopathological Significance and Potential Drug Target of CDKN2A/p16 in Endometrial Carcinoma. *Sci. Rep.* **5**, 13238; doi: 10.1038/srep13238 (2015).

## Figures and Tables

**Table 1:** Basic characteristics of the included studies.

**Study**	**Country**	**No. of EC tissue/control**	**Methods**	**Methylation site**	**CDKN2A expression**
Zhang *et al.* 2011^28^	China	35/15	MSP	Promoter, CpG islands	−
Zhou *et al.* 2009^29^	China	38/20	MSP	Exon 1	−
Guida *et al.* 2009^30^	Italy	40/25	MSP	Promoter, CpG islands	−
Ignatov *et al.* 2008^22^	Germany	54/ND	MSP/IHC	Promoter, CpG islands	+
Suehiro *et al.* 2008^21^	Japan	103/27	MSP	Promoter, CpG islands	−
Yang *et al.* 2006^26^	China	134/ND	MSP	Promoter, CpG islands	−
Yun *et al.* 2004^31^	China	62/10	MSP	Exon 1	+
Semczuk *et al.* 2003^23^	Poland	11/ND	MSP/IHC	Promoter, CpG islands	+
Wang *et al.* 2002^24^	China	36/12	MSP	Exon 1	−
Chao *et al.* 2000^27^	China	48/14	MSP, RT-PCR	Exon 1	+
Zhao *et al.* 2000^32^	China	38/8	MSP, RT-PCR	Exon 1	+
Wong *et al.* 1999^25^	Hong Kong	49/ND	MSP	Promoter, CpG islands	−

ND: not determined.
